# Cultural orientations and information systems success in public and private hostitals: preliminary evidences from Italy

**DOI:** 10.1186/s12913-018-3349-6

**Published:** 2018-07-16

**Authors:** Luigi Lepore, Concetta Metallo, Francesco Schiavone, Loris Landriani

**Affiliations:** 1 Department of Law, University of Naples Parthenope, Naples, Italy; 2 Department of Science and Technology, University of Naples Parthenope, Centro Direzionale –Isola C4, 80143 Naples, Italy; 3 Department of Management Studies & Quantitative Methods, University of Naples Parthenope, Naples, Italy; 4 Department of Strategy and Management, Paris School of Business, Paris, France; 5 Department of Management, Accounting and Economics, University of Naples Parthenope, Naples, Italy

## Abstract

**Background:**

The effective adoption and use of digital and computerized systems and records in hospitals are crucial for increasing the overall quality, safety and outcomes of any national health community. Prior research found that hospitals’ dominant cultural orientation affects the adoption of new technology. However, the organizational culture of hospitals can greatly vary between public and private hospitals. Thus, the ownership type of the hospital is likely to affect, to some extent, the aforementioned relationship between culture and information system success. The present article focuses in detail on this issue and attempts to answer the following research question: which cultural orientations are promoting information system success in public and private hospitals?

**Methods:**

The authors develop and test two hypotheses about this relationship via two regression approaches (single-level and multi-level). The authors collected data from 172 respondents—clinicians and non-clinicians—working in two (one public and one private) hospitals in Campania, one of the largest regions in Italy.

**Results:**

The findings of this study show clear differences between private and public hospitals. First, a dominant cultural orientation that emphasizes flexibility values (clan and adhocracy cultures) positively influences information systems success in terms of individual impact. Second, the influence of a clan orientation on individual impact is stronger in the public hospital. Third, the influence of an adhocracy orientation is stronger in the private hospital. Overall, the type of ownership—either public or private—of these healthcare organizations affects the link between cultural orientations and IS success.

**Conclusion:**

Managers of private hospitals should offer to their employees the opportunity to adopt and implement new information systems processes driven by openness towards the external environment in order to benchmark and learn from what was done previously in other organizations. Managers of public hospitals should set up human resource management practices, knowledge creation mechanisms, and internal communication capable of generating a friendly learning environment for their employees when adopting new technology.

## Background

The literature on IS in healthcare agrees that contingent factors, such as OC, are often overlooked or underemphasized in evaluating the success or failure of a technology adoption (e.g., [1–3]), suggesting the need for more research on health information systems success that focuses on OC.

Information system success (IS) is a complex construct because the definition and measurement of success can depend on the setting, objectives, and stakeholders of the organization [2]. The success “is critical to our understanding of the value and efficacy of IS management actions and IS investments” ([4], p. 10). The effective adoption and use by hospitals of digital and computerized systems and records are crucial for increasing the overall quality, safety and outcomes of any national health community (e.g., [5–8]). Thus, IS can lead hospitals and other healthcare organizations to effectively react to the various on-going technology-based changes currently occurring within this industry. The framework most frequently used to measure IS is the DeLone and McLean [9, 10] model.

They consider six dimensions to define success in the adoption and use of IS: system quality; information quality; information use; user satisfaction; individual impact; and organizational impact. The review by Van Der Meijden and co-workers [2] emphasizes that these success dimensions can also be used for evaluating health information technology (HIT). Indeed, several studies within health research analysed the success of HIT by using the DeLone and McLean model (e.g., [11–13]).

Among the various dimensions of IS that DeLone and McLean outline, we pay particular attention to individual impact. The focus on the individual impact dimension emphasizes the extent to which information can influence the tasks that are performed by the user, thus changing work practices ([14], p. 133). Berg [15] reports that success can mean users appreciate the use of the system, and thus, their perceived benefits can play an important role in determining success. The IS should provide relevant information to help users to best perform their jobs in terms of efficiency, task accomplishment, teamwork, and the quality of decision-making [16]. Prior studies in IS research also stress organizational characteristics’ impact on IS. Several authors investigated the relationship between organizational culture (OC) and technology (for a review, see [4]). However, research is less focused on the effects of specific cultural values on new technology adoption and outcomes, such as the successful implementation of IS [4]. Within these few studies, scholars have shown that some cultural values emphasize a greater individual propensity to adopt a new technology and thus to influence IS (e.g., [1, 17]).

With specific reference to IS in healthcare, a great deal of research reports that contingent factors, such as OC, are often overlooked or underemphasized in evaluating the success or failure of a technology adoption (e.g., [2, 6, 13, 14]). OC is an important value-adding strategy for healthcare organizations [18]. For instance, prior research found that OC oriented towards participation and commitment reduces medical errors in hospitals [19] and increases job satisfaction and perceived clinical effectiveness [20]. This evidence suggests the need for more research on the relationship between health IS and OC.

This concept relates to what is most valued: the dominant leadership styles, the language and symbols, procedures and routines, and the definitions of success that make an organization unique [21]. These values are considered to be central to understand an organization’s culture [22], and thus, the analysis of OC has typically focused on such values.

The literature proposes many constructs with which to measure organizational culture. Among these models, the competing values framework (CVF) [23] is sometimes preferred for evaluating the organizational culture because it contemporarily considers several competing values, whose combination gives rise to four types of cultural orientations that coexist in the organization. Therefore, within the organization the several types of cultural orientations emerge from workers’ preferences regarding the dominant values. In other words, the framework reflects the realistic consideration that every organization has its own combination of different types of cultural orientations.

The CVF is a useful established tool for understanding OC in healthcare settings (e.g., [24–26]). Based on important CVF research contributions that propose enhancements to the original framework, Cameron and Quinn [21] presented the Organizational Culture Assessment Instrument (OCAI), an instrument that assesses the overall culture profile. This framework is based on two-dimensional space that reflects different value orientations [21, 23]: flexibility versus control and internal orientation versus external orientation. The flexibility versus control dimension stresses the differences between organic and mechanistic forms [27], emphasizing values such as spontaneity, change, and dynamism with respect to values of stability, order, and control. The internal orientation versus the external orientation dimension refers to the organization’s choice between focusing on internal dynamics (in terms of maintaining and improving the existing organization) and interaction with the external environment. These two dimensions give rise to four types of cultural orientations [21]:

1. A clan culture concentrates on internal dynamics and values flexibility and discretion, emphasizing a humane work environment, teamwork and employee development, as customers are considered partners [21]. “The clan culture is typified by a friendly place to work where people share a lot about themselves. It is like an extended family” ([21], p. 37).
2. An adhocracy culture focuses on external dynamics and flexibility, with key values that emphasize discretion, creativity and risk taking. These organizations are innovative, adaptable and are “characterized by a dynamic, entrepreneurial, and creative workplace” ([21], p. 3).
3. A hierarchy culture refers to Weber’s hierarchy or bureaucracy. This type of OC concentrates on internal dynamics and control, emphasizing a formal and structured workplace with a clear authority, standardized rules and procedures, and control and accountability mechanisms [21].
4. A market culture is characterized by values of stability and control as well as external orientation. Market-oriented organizations focus on transactions with the external environment and put emphasis on a results-oriented workplace and aspects such as productivity and competitiveness ([21], p. 36).

Another relevant variable to consider in order to better understand the relationship between OC and IS in this context is the ownership type (public vs. private) of healthcare organizations. Indeed, this variable is, to date, still unexplored in the literature. The distinctions between public and private organizations have been studied in the management and organizational literature for a long period of time [28]. With regard to cultural profiles and orientations, prior research reports that public servants and private managers hold very different sets of values [29]. In line with this evidence, a rich body of studies in the healthcare literature also stresses the various differences between private and public hospitals, for instance, in terms of quality of care, patient satisfaction, organizational climate, performance and costs [30–32]. All these findings suggest that the ownership type of the hospital is also likely to affect to some extent the aforementioned relationship between OC and IS.

Therefore, the research question of this article is as follows: which cultural orientations are promoting IS in public and private hospitals?

This research question would contribute to the rising literature about the impact of culture on the performance of hospitals worldwide. In some countries, such as Turkey [33], the cultural evolution of public hospitals towards market competition is still slow and makes hard for these organisations to achieve positive performance.

## Research model

Within IS literature, the technology acceptance model (TAM) is one the main theories explaining the acceptance and adoption of new technology by individuals and/or organizations [34–36]. The model reports the intention to use, for instance, a new PC or tablet emerges when the individual finds it useful and easy to use. If these conditions are satisfied, then the user will adopt the new technology. The TAM model provides the basic assumptions of the present article since its outcome is, at the end, IS.

Although the perception of IS can also change between different countries [17], prior research stresses the link between a flexible OC and the successful adoption of IS. An open and collaborative cultural orientation generally has a positive effect on readiness to change, characterizing more favourable outcomes in terms of IS [2, 18, 37], OCs characterized by flexibility and supportive climates affect the successful implementation of a new technology better than mechanistic organizations characterized by control do. Therefore, organizations with cultural archetypes characterized by higher flexibility, limited authority, and poor hierarchical rules and procedures are more prone to adopt an innovation technology and, in general, to change. In this way, some scholars [38, 39] showed that for the successful implementation of HIT, such as computerized provider order entry, hospitals must have a collaborative organizational culture that emphasizes teamwork and participative decision-making. Therefore, flexibility-oriented cultures enhance innovation because flexibility is associated with values such as creativity, freedom, and a risk-taking attitude, whereas cultures that stress stability and control can inhibit innovation [11, 39]. In fact, the bureaucratic and mechanistic nature of the OC appears to be an obstacle to change and, in particular, to the adoption of innovation technology [40, 41].

In line with the literature, we expect that a cultural orientation that emphasizes flexibility values (clan and adhocracy cultures of the CVF) positively influences IS in terms of individual impact. Therefore, we hypothesize the following:

*H1: Flexibility-oriented cultures (clan and adhocracy culture) positively influence IS compared with control-focused cultures.*

Moreover, we expect that the ownership type of healthcare organizations, in terms of public or private hospitals, affects the relationship between flexibility-oriented cultures and IS. In fact, ownership status can play a relevant role in explaining the behavioural differences between public and private organizations in the health sector [42, 43]. For example, the decision to implement HIT in public hospitals could be the result of the size of the budget, which is often larger than that of private hospitals [44].

There has been a significant growth in studies comparing public and private healthcare organizations aimed at investigating whether one type of ownership is more effective than others in delivering certain outcomes (e.g., [23, 45, 46]). Nevertheless, relatively little is known about the relationship between ownership type and organizational performance, mainly in terms of IS, because the results of the available empirical studies show contrasting findings. For instance, some research found that public hospitals were relatively more efficient than private hospitals (e.g., [47, 48]). In contrast, other studies found the opposite results, revealing better performance in private hospitals than that in public organizations (e.g., [49, 50]). Other research fails to show differences based on ownership status (e.g., [51–53]). Moreover, Sloan’s [51] research regarding the effects of ownership type on hospital behaviour has emphasized that only a few scholars have investigated the relationship between ownership and the adoption of innovation technology by distinguishing between public and private organizations (e.g., [54–58]).

Finally, several studies have stressed the importance of organizational characteristics (such as OC) in the relationship between ownership status and performance (e.g., [59]). Therefore, research on public-private differences requires more sophisticated research designs in which other variables should be included [60, 61]. Thus, we hypothesize the moderating effect of ownership type (public or private) on the relationship between flexibility-oriented cultures and IS. Public and private hospitals differ with regard to their mission. Public hospitals are typically aimed towards serving the community, while private hospitals seek to return a profit to their shareholders [62]. As such, private hospitals are goal-oriented, motivated by an external push to improve their performance. Public hospitals, however, are more process-oriented and do not have the same strong, external push encouraging them to improve their performance. Thus, assuming that the IS usage is aimed towards improving the individual and organizational performance, the presence of external or internal orientation that respectively characterizes private and public hospitals may differentiate the way flexibility-oriented cultures influence IS. Therefore, we hypothesize the following:

*H2: The ownership type moderates the relationship between cultural orientations and IS, such that the clan fits better with the public hospitals, whereas the adhocracy fits better with private hospitals.*

**Fig. 1:**
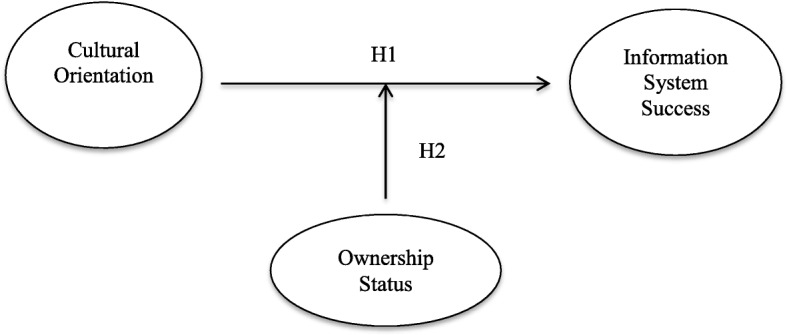
Research Model

Overall, this study is aimed to analyse how the cultural orientation of a hospital relates to IS, deepening the importance of the role of ownership status as a moderator variable (Fig. 1).

## Methods

### Setting

This study was conducted in two hospitals between October and December 2016. The two hospitals have very similar characteristics, for example, in terms of size and in terms of geographical location: they have the same number of beds and both operate in a territory characterized by a high demand for health services, i.e., the Campania Region in southern Italy, due to the high population density. The most important difference is the ownership type: the first is a public non profit hospital, and the second is a private for-profit organization. Campania is the third largest region in Italy, with approximately 9.6% (5.8 million) of the national population, and it has the second highest public debt in the healthcare sector.

Clinicians and non-clinician staff in these hospitals have been using a specific HIT application known as Electronic Health Record for about 2 years. This application is a medical information system that is substituted for the traditional paper medical record and refers to a comprehensive record of a patient health care history in digital format. The record contains all of a patient’s health information (medical records, demographics, laboratory data, medication and other important medical information) and is accessible electronically by healthcare providers through a mobile device.

### Sample and data collection

We conducted a survey using a questionnaire sent to both clinicians and non-clinicians at the aforementioned hospitals. The questionnaire was divided into two sections. The first was intended to capture the profile of the survey respondents (age, gender, education level, and IT experience), the information systems used and the perceived impact of IS on the respondents’ work performance. The second section contained the CVF/OCAI questions necessary for categorizing the user’s cultural orientation.

First, we conducted a pilot test to verify and validate the measures used, obtaining feedback from IS users from both hospitals and IS scholars. The findings of the pre-test highlighted the reliability and consistency of the scales used. Then, we administered a total of 300 questionnaires to medical and non-medical staff at the hospitals, and we received a total of 180 complete questionnaires – 110 from the public hospital (representing a return rate of 73.33%) and 70 from the private hospital (46.66%). To minimize data-entry errors, all the collected data were checked for consistency. This check resulted in 172 valid responses (103 from the public hospital and 69 from the private hospital).

### Variables

#### Information systems success (IS)

We measure our dependant variable, the IS, by considering the individual impact, which is the extent to which information can influence the tasks that are performed by the user, changing work practices and improving her/his individual performance. More generally, the individual impact is the dimension of the IS of the DeLone and McLean [10] model that refers to success perceived by the user regarding individual performance improvement resulting from IS usage. In particular, our dependant variable measures “the degree of success of application software in terms of (i) improving the user’s quality of work, (ii) making the end user’s job easier, (iii) saving the end user time, and (iv) helping fulfil the needs and requirements of the end user’s job” ([63] p. 66). The variable was measured by adapting Etezadi-Amoli and Farhoomand’s [63] user performance four-item scale.

#### Cultural orientation (CO)

To assess CO, the opinions of human resources, who are the “bearers” of the corporate culture, are taken into account in relation to 6 dimensions of OC: 1. the dominant characteristics of the organization (e.g., dynamic, formal, familiar); 2. leadership style (e.g., paternalistic, aggressive, coordinator); 3. human resource management (e.g., support for group work vs. individual work); 4. organizational glue (e.g., loyalty, innovation, result, rules); 5. strategic emphasis (e.g., human resources, creativity, market dominance, efficiency and control); and 6. the criteria of success (e.g., staff, product leadership, competition, efficiency).

For each of the six aspects mentioned above, the OCAI questionnaire offers respondents a set of four possible descriptions of an organization, each corresponding to a different type of culture (Clan, Adhocracy, Hierarchy and Market). For each set of descriptions, the clinician and non-clinician respondents had to allot 100 points to the descriptions that best matched his or her perception. The cultural type that received the highest score was outlined as the dominant CO. To define the independent variables used in our regressions, we named the four possible CO’s as *Clan_CO, Adhocracy_CO, Market_CO* and *Hierarchy_CO*. Each respondent perceives the hospital where she/he works as an ensemble of Clan, Adhocracy, Hierarchy and Market, but often only one of these archetypes prevails (the dominant CO).

By aggregating the scores provided by all the respondents operating in each hospital, we are able to define the two cultural models for the two hospitals. Similar to the CO of each respondent, the cultural model for each hospital is the result of a mix of the competing values of each cultural archetype (Clan, Adhocracy, Market, and Hierarchy), but often, also at the organizational level, one archetype prevails. Such an archetype is the dominant cultural orientation of the hospital. Several empirical studies in various fields have tested the validity and reliability of the CVF/OCAI test (e.g. [11, 24, 27, 28]).

#### Ownership type public or private (Pub_or_Pri)

Ownership type is a dummy variable used as a moderator that indicates the public or private hospital. This variable takes the value of 1 if the respondent works in the private hospital, 0 if otherwise.

#### Control variables

As the IS literature recommends, in order to better evaluate the effect of the independent variables on the dependent variable, several questions were used as control variables: age, gender, educational level and the IT experience of the respondents. IT experience is a continuous variable, representing the years of IT use.

### Statistical analysis

To assess the effects of the CO on IS in public and private hospitals, we tested our hypotheses using single-level (8 models) and multi-level regressions (8 models). In each single-level regression, we consider *IS* to be the dependent variable and one of the four possible CO’s (*Clan_CO, Adhocracy_CO, Market_CO* and *Hierarchy_CO)* to be the independent variable. We preferred to include each of the possible CO’s in separate regression models in order to avoid multi-collinearity problems among the independent variables.

To understand whether the effects differ across public and private hospitals, we also incorporated the dummy variable *(Pub_or_Pri)* and the interaction terms for group membership (Clan_CO*Pub_or_Pri; Adhocracy_CO*Pub_or_Pri, Market_CO*Pub_or_Pri and Hierarchy_CO*Pub_or_Pri). In this way, it was possible to understand whether the intercepts and slope coefficients differ across groups. We also implemented regressions with cluster-robust standard errors to correct for clustering within groups, but the results (not reported) are very similar. Moreover, as a robustness check, we implemented a multivariate hierarchical regression (two-level regression with random intercepts and slopes) in which the respondents are nested within their hospital. The analysis of the intraclass correlation coefficient also suggests using the multilevel approach. This statistical method explicitly takes into account the hierarchical structure of the data and helps us to understand whether variables at the organizational level, such as the hospital ownership type, play a role in the relationships analysed.

## Results

**Fig. 2:**
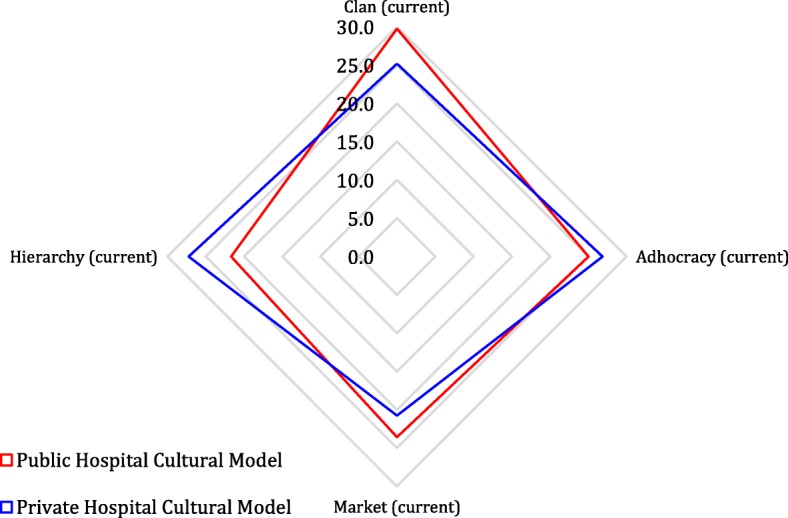
Public and Private Hospitals’ Cultural Model

Table 1 provides the descriptive statistics for the variables. As is also shown in Fig. 2, the dominant CO for the public hospital is the clan; this finding means that the respondents place more emphasis on aspects such as concern for people and collegiality. Other archetypes have lower values; in particular, the lowest level of hierarchy means that the respondents perceive a slight orientation towards rules, stability and control. In contrast, the dominant CO for the private hospital is the hierarchy; however, the adhocracy and clan orientations also show important values, whereas the values for the market orientation are lower. These findings mean that authority, rules and procedures are the principal mechanisms of coordination in the private hospital and that this perception prevails over the others, though values of clan and adhocracy are nevertheless perceived. The low scores in the market quadrant for both public and private hospitals may indicate that the respondents care much less about competitive strategies and profitability than they do about the people side of the organization.

**Table 1:** Descriptive statistics

	Public Hospital		Private hospital		Full sample	
Variables	Mean	Std Dev	Mean	Std Dev	Mean	Std Dev
IS	3.323	1.880	4.138	1.787	3.650	1.881
Clan_CO	29.742	7.745	25.174	9.240	27.909	8.648
Adhocracy_CO	25.021	4.578	26.858	5.775	25.758	5.156
Market_CO	23.572	5.144	20.760	6.521	22.444	5.883
Hierarchy_CO	21.664	5.288	27.209	7.344	23.888	6.750
Gender	0.592	0.494	0.536	0.502	0.570	0.497
Age	45.932	13.888	40.826	11.558	43.884	13.209
Educational_level	2.388	0.910	2.594	0.773	2.471	0.861
IT_experience	15.796	3.414	9.348	5.670	13.209	5.457
N. observations	103		69		172

The people working in the hospitals range from 21 to 84 years of age, and the average age is 43.9 years. With regard to gender, out of 172 workers, 98 are male (56.9%) and 74 are female (43.1%). The data also show that hospital staff is highly educated. Indeed, 102 of them had a bachelor’s degree (59.3%), and seven had a master’s degree or PhD (4.06%). With regard to IT experience, workers are sufficiently experienced with an average of 13.2 years and a range from 1 to 21 years.

The overall results of the single-level regressions consist of 8 models shown in Table 2.

**Table 2:** Single level regression models

Single-level								
Dependent variable IS								
Variables	Model 1	Model 2	Model 3	Model 4	Model 5	Model 6	Model 7	Model 8
Clan_CO	0.1099175*** 7.78	0.15903148*** 7.86						
Clan_CO*Pub_or_Pri		−0.07331773* − 2.47						
Adhocracy_CO			0.0776647** 2.72	0.0008058 0.02				
Adocracy_CO*Pub_or_Pri				0.1262711* 2.25				
Market_CO					−0.1335223*** − 6,07	−0.1499334*** − 4-59		
Market_CO*Pub_or_Pri						0.0382324 0.83		
Hierarchy_CO							−0.1258365*** −6.52	−0.1842832*** −6.03
Hierarchy_CO*Pub_Pri								0.0495197 1.20
Pub_or_Pri		3.3059325*** 3.68		−2.798.527 −1.88		−0.6143535 −0.58		0.3369997 0.33
Age	−0.0038931 − 0.34	−0.00490541 − 0.42	−0.0221063 − 1.66	−0.0192218 − 1.41	−0.0156675 − 1.29	−0.0162451 − 1.30	−0.0132182 − 1.11	−0.0239061* − 2.06
Gender	−0.1177421 − 0.47	−0.14326926 − 0.61	0.10037 0.35	0.1610175 0.56	0.0917031 0.35	0.0439377 0.16	−0.0431238 − 0.17	−0.0227898 − 0.09
Educational_level	0.3255974* 2.19	0.344109* 2.42	0.37949* 2.19	0.2951138 1.70	0.3785616* 2.40	0.3749512* 2.36	0.3996783* 2.57	0.4281056** 2.80
IT_experience	−0.0771391** − 2.84	0.00354347 0.11	− 0.0322267 − 1.04	−0.0109561 − 0.29	−0.0240426 − 0.84	−0.0086911 − 0.25	−0.081359** − 2.85	0.0091786 0.28
Obs	172	172	172	172	172	172	172	172
R2	0.3210	0.4032	0.1127	0.1478	0.2416	0.2467	0.2622	0.3531
R2 adjusted	0.3006	0.3778	0.0860	0.1115	0.2188	0.2146	0.2400	0.3255
F-stat	15.70	15.83	4.22	4.07	10.58	7.67	11.80	12.79
Prob > F	0.0000	0.0000	0.0012	0.0004	0.0000	0.0000	0.0000	0.0000

*P-value* (Significance) legend: * *p* < 0.05; ** *p* < 0.01; *** *p* < 0.001. T-statistics are provided under the estimated coefficient

Models 1, 3, 5 and 7 assume no difference between public and private hospitals. Models 2, 4, 6 and 8 assume that the intercepts and slope coefficients differ across groups. The results of Model 1 confirm hypothesis H1, i.e., that *Clan_CO* positively influences the *IS*. The significant negative coefficient of Clan_CO*Pub_or_Pri in Model 2 confirms hypothesis H2 with regard to *Clan_CO*. This finding indicates that the performance of users in the private hospital benefits less from the clan orientation than that of the users in the public hospital. Specifically, each point of the *Clan_CO* is worth approximately 0.073 less in terms of *IS* for a private hospital user than it is for a public hospital user. Models 3 and 4 show the results regarding H1 and H2 with reference to *Adhocracy_CO*. An adhocracy orientation positively influences *IS*, and the positive significant coefficient of the interaction term means that the adhocracy orientation has a larger effect on *IS* in the private hospital than it does in the public hospital. The more the adhocracy orientation increases, the more the *IS* increases for the users in both the public and private hospitals. However, the increase (slope) is much greater for users in the private hospital. The results of Models 5 and 7 represent further evidence that H1 is confirmed: *Market_CO* and *Hierarchy_CO* negatively influence *IS*. In fact, the market and hierarchy orientations are characterized by a low level of flexibility values. Models 6 and 8 show that the interaction terms for *Market_CO* and *Hierarchy_CO* are not significant.

Moreover, our models also show the effect of control variables (age, gender, educational level, and IT experience) on the dependent variable. In particular, findings show that educational level positively affects IS, while models 1 and 7 show that IT experience negatively influence *IS*.

The latter result is probably because workers more accustomed to working with a certain technology find it more difficult to adapt to the use of a new technology (such as Electronic Health Record).

**Fig. 3:**
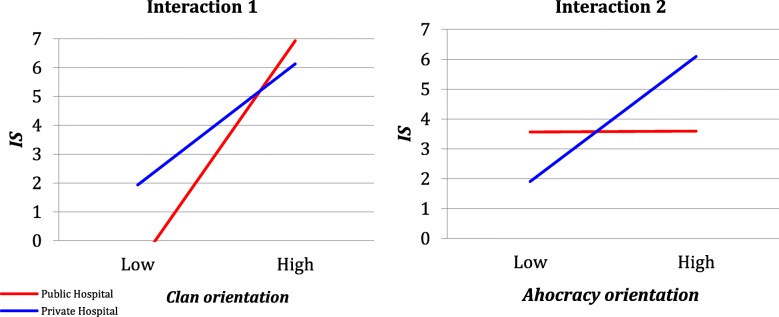
The interaction effects

The interaction effects for the clan and adhocracy orientation are shown in Fig. 3.

The results of the multivariate hierarchical regressions are shown in Table 3. Models 1, 3, 5, and 7 contain only the user-level variables and serve as baselines for assessing the degree to which adding a group variable in models 2, 4, 6, and 8 improves the fit of the models. The intraclass correlation coefficients ranged from 0.02 to 0.18, indicating that 2 to 18% of the scale variance is between hospitals. The *Clan_CO* appears to show larger differences across hospitals compared to the other culture types. The results of the hierarchical regressions are very similar to those of the single-level approach as shown in Table 3. Thus, analogous conclusions can be obtained: *Clan_CO* and *Adhocracy_CO* positively influence *IS*, while *Market_CO* and *Hierarchy_CO* negatively affect *IS*. With regards to the interactions, the findings also confirm that 1) *Clan_CO* has a greater effect on *IS* in the public hospital than it does in the private hospital, and 2) *Adhocracy_CO* has a larger effect on *IS* in the private hospital than it does in the public organization. Log-likelihood ratio tests suggest opting for the single-level approach.

**Table 3:** Multi-level regression models

Multiple-level								
Dependent variable IS								
Variables	Model 1 Varying intercept	Model 2 Intercept and slope coefficient differ	Model 3 Varying intercept	Model 4 Intercept and slope coefficient differ	Model 5 Varying intercept	Model 6 Intercept and slope coefficient differ	Model 7 Varying intercept	Model 8 Intercept and slope coefficient differ
*Individual-level*								
Clan_CO	0.1204917*** 9.07	0.1597953*** 8.26						
Adhocracy_CO			0.0771315** 2.76	0.007459 0.18				
Market_CO					-0.1337026*** -6.21	-0.1562548*** -4.96		
Hierarchy_CO							-0.1537486*** -8.36	-0.1880278*** -6.45
Age	-0.0161782 -1.47	-0.0082306 -0.74	-0.0283078* -2.15	-0.022354 -1.67	-0.0204219 -1.70	-0.0194278 -1.60	-0.0298436** -2.65	-0.0282873* -2.52
Gender	-0.1427667 -0.62	-0.161546 -0.72	0.0822964 0.30	0.1479806 0.54	0.0756863 0.30	0.0197324 0.08	-0.0684146 -0.29	-0.0450167 -0.19
Educational_level	0.3089588* 2.26	0.3565551** 2.62	0.3807205* 2.27	0.3104131 1.84	0.3847769* 2.53	0.3902247* 2.56	0.3979017** 2.82	0.4477889** 3.06
IT_experience	0.0038446 0.12	0.0178511 0.58	0.0017332 0.05	0.0001423 0.00	-0.0001292 -0.00	0.0056921 0.16	0.0181211 0.57	0.0283402 0.88
*Group-level*								
Pub_or_Pri		3.269601*** 3.49		-2.666175 -1.81		-0.8577904 -0.78		0.170129 0.16
Clan_CO*Pub_or_Pri		-0.0744968** -2.63						
Adocracy_CO*Pub_or_Pri				0.1192239* 2.19				
Market_CO*Pub_or_Pri						0.045347 1.02		
Hierarchy_CO*Pub_or_Pri								0.0541163 1.37
Random Effect								
Variance Component								
Intercept; Slope	0.3367933	0.104813; 6.14e-15	0.1300905	0.0682498; 4.49e-21	0.1193571	0.1230823; 7.02e-14	0.4961523	0.129275; 1.12e-15
Wald X2 (Df)	97.09 (5)	111.64 (7)	18.18 (5)	24.74 (7)	50.84 (5)	52.26 (7)	84.06 (5)	90.29 (7)
Prob > X2	0.0000	0.0000	0.0027	0.0008	0.0000	0.0000	0.0000	0.0000
LR test (vs. linear reg.)	0.0001	0.2296	0.0609	0.6993	0.0510	0.2703	0.0000	0.1887
Var (Residual)	2.094066	2,010941	3.007963	2.940197	2.564107	2.546349	2.196045	2.170528
ICC	0.1385491	0.0495393	0.0414558	0.0226861	0.0444787	0.046108	0.1842926	0.0562113
AIC	639.1471	634.754	697.5377	698.2673	670.2606	675.1609	648.5057	648.2463
BIC	664.3271	669.3765	722.7177	732.8897	695.4405	709.7833	673.6857	682.8688
Obs	172	172	172	172	172	172	172	172

*P-value* (Significance) legend: * *p* < 0.05; ** *p* < 0.01; *** *p* < 0.001. *Z-statistics* are provided under the estimated coefficient

## Discussion

This paper aims to understand the influence of cultural orientation on the perceived individual performance improvement resulting from IS usage in public and private healthcare organizations. The results highlight the relevant role of flexibility in explaining IS and the importance of the ownership type (public or private) as a moderating factor in the relationship between the cultural orientation and IS.

Our hypotheses have been confirmed. In particular, the positive and significant relationship between a clan/adhocracy orientation and perceived individual performance improvement (H1) confirms the assumption that organizations with cultural archetypes characterized by higher flexibility, little authority, poor hierarchical rules and procedures, and higher autonomy of human resources better utilize technology and, in general, innovations and change [17]. In fact, our findings reveal that the dominant cultural models affecting IS are the clan and the adhocracy in public and private hospitals, respectively. As highlighted above, important differences between private and public hospitals appear when considering the internal or external focus of these two cultural archetypes. In fact, the clan, which is internally oriented, is the most effective cultural orientation in terms of perceived individual performance improvement for public hospitals, while the adhocracy, which is externally focused, is the most effective cultural orientation in terms of perceived individual performance improvement for private hospitals.

These findings can be explained in light of the following considerations. According to Van der Wal, De Graaf and Lasthuizen [29], from an institutional perspective, public and private organizations mainly differ in their mission and values. Public organizations operate in a cartel condition; they cannot measure the output by the market price, and they primarily have social goals and a non profit orientation. Thus, unlike private, for-profit organizations, public organizations do not have an external motivation to improve their performance because they are normally not externally focused. Second, from an organizational perspective, public organizations are characterized by self-referentiality [64], the absence of incentives, fixed salaries, a bureaucratic process orientation (more than a goal orientation), and routine activities [19]. Furthermore, from a cultural perspective, in public organizations, there is a low level of employee engagement because organizational performance does not impact individual benefits, above all in the healthcare context [29]. This condition of employees induces a closure of public organizations and discourages a stakeholder orientation [29]. In fact, some previous studies have highlighted the possibility that the clan-oriented cultural approach is predominant in the public context because the organization is perceived as “extended family” [65], meaning a traditional place [66] able to reinforce commitment and intrinsic motivation [67] and where workers are not motivated by salary [68, 69].

In summation, the clan (internally oriented) fits better with the public sector because it is based on the sharing of common values (even if often converting by the daily routine) without the need to search for entrepreneurial opportunities [56], whereas the adhocracy fits better with private organizations, which, by nature, are more adaptability, dynamic and risk-oriented.

Finally, from an operational point of view, the main differences between public and private hospitals are characterized by the supply, production and sales processes [70], which inevitably impact the information system management [56]. Regarding the supply process, in the public sector, each purchase must be closed after a public competition or tender, and this determines very long delivery, as opposed to the process in the private sector, which operates on the market and can immediately dispose of assets (organizational interdependences and red tape). The production phase in public hospitals is usually characterized by the reduction in interventions or examinations not strictly necessary and for to the short duration of hospitalizations, with risks for patients (evaluating hardware and software). Moreover, the sales phase in public hospitals is characterized by the absence of market prices (which are very high in the private hospitals) and by the presence of social tariffs to guarantee the protection of a fundamental right (planning process). This result causes poor performance for public hospitals and lower managerial responsibility (organizational level of the data processing manager).

The afore mentioned characteristics of public and private hospitals are the result of the evolution of the healthcare industry, which currently tends to be more market oriented, focusing on efficiency and service quality (effectiveness). In recent decades, in fact, some elements of the public healthcare sector had to change to a free market system, increasing patient safety and demanding better service than what old-fashioned state hospitals could supply. At present, public hospitals have had to prepare for competition and better respond to the needs of clients. In a similar context, the use of technology supports this change in order to increase flexibility, dynamic processes and consumer orientation. In doing so, the information system is perceived by users as the way to improve personal and managerial performance. From a cultural perspective, public healthcare organizations have been pushed to move – gradually and with difficulty – from clan and hierarchical models towards adhocracy and market models [71]. This process, unfortunately, is still in progress. Public organizations in the healthcare sector and in other public sectors continue to be internally focused. Therefore, the healthcare system has become more expensive, slower, and more rigid more rapidly than it has changed, owing to the innovation processes as run by legislators. It seems obvious that a clan orientation is currently still more effective in increasing perceived individual performance improvement in public hospitals than an adhocracy orientation, while the latter has a larger influence on perceived individual performance improvement in private hospitals [45, 47, 48].

Finally, the negative and significant relationships between the market/hierarchy orientation and perceived individual performance improvement corroborate the assumption that organizations characterized by strong stability, where authority, rules and procedures are the principal mechanisms of coordination, experience far more difficulties in the implementation of new technologies and, in general, in change or innovation. These considerations clarify why our findings show that the influence of cultural orientation on perceived individual performance improvement is moderated by the public or private ownership of the hospital only for the clan and adhocracy archetypes, while for the market and hierarchy archetypes, there is no significant interaction effect. This finding means that there are no important differences in the ways in which market and hierarchical orientations influence perceived individual performance improvement across public and private hospitals [39, 45].

## Conclusions

Our research is aimed at investigating the effect of the ownership type of healthcare organizations on the relationship between cultural orientation and IS in terms of individual impact. The findings from 172 responding clinicians and non-clinicians point out clear differences between private and public hospitals. In general, the results show that for healthcare organizations, the OC in IS usage is an important issue. First, a dominant cultural orientation that emphasizes flexibility values (clan and adhocracy cultures) positively influences IS in terms of individual impact. Second, the influence of a clan orientation on individual impact is stronger in the public hospital. Third, the influence of an adhocracy orientation is stronger in the private hospital. Overall, the public-private ownership within healthcare organizations affects the link between cultural orientations and IS success.

These findings could stem from the partial realization of the reform process of the healthcare sector, which has tried, without complete success, to push public healthcare organizations to transition from being internally focused and control-oriented organizations to being externally focused and flexibility-oriented organizations. Flexibility-oriented cultural values (clan and adhocracy cultures) are important for IS in both public and private hospitals. Therefore, some managerial implications emerge from our results. Healthcare managers should encourage these flexibility-oriented cultural orientations for achieving better performance in term of IS, adopting human resource management practices that promote values such as goal sharing, the distribution of authority or cohesion among workers. Managers should favour a friendly environment, where colleagues have more in common and share commitment, enforce dynamicity in the workplace and foster innovation.

In particular, private healthcare management should further strengthen adhocracy values, such as learning, improvement, experimentation, and the freedom for workers to make their own professional decisions. Thus, private hospitals should design training processes for IS users driven by openness towards the external environment or benchmark in order to learn what was done previously in other organizations using the same or similar IS. For instance, managers could promote the participation of some employees in conferences and workshops with external experts. Moreover, executives and staff should have a more vital and dynamic working environment because they perceive a healthcare sector that encourages more personal freedom in their field of specialization, as well as more personal responsibility and individual initiative.

Additionally, public healthcare managers—whose structures still maintain a lower external orientation—should favour a clan orientation, which may be a very convenient and properly fitting culture type when workers cooperate to try to heal or look after people, without underestimating the necessity of simultaneously fostering adhocracy values because the in-progress innovation process leads to a greater external orientation. Therefore, managers of public hospitals should set up human resource management practices, knowledge creation mechanisms, and internal communication able to generate a friendly environment of learning for their mployees engaged in the use of new IS. For instance, in-house informal seminars—or similar events—could be frequently arranged to foster a clan-oriented organizational culture. During these meetings, the hospital health professionals and administrative staff could become aware of (and probably also contribute to the definition of) the IS strategy, challenges and goals pursued by their organization. Moreover, they could also learn in these meetings how to use the new IS adopted by the hospital.

This study has some limitations that must be acknowledged and taken into account in future research. The first limitation concerns common method biases, a potential problem in behavioural research that arises from using self-report measures. The use of self-report measures may be subject to bias, which distorts and exaggerates the causal relationship between the independent and dependent variables. However, for studies concerning people’s perceptions and feelings, the data are usually based on self-assessment. The second limitation is that this study relied on cross-sectional data, thus raising concerns about the causality of the research results. People’s perceptions can change over time; it is important to measure these quantities at several points of time. Future research might attempt to replicate our findings with research designs based on longitudinal data collection. Another limitation stems from the sample, which included all categories of workers, including both clinicians and non-clinicians. Future research should also analyse whether this distinction between categories of workers within healthcare organizations has implications for the results of this study. Moreover, we tested the hypotheses in only two hospitals with very similar characteristics (such as, size and geographical location). Although this study is exploratory, caution must be exercised in generalizing the findings. Therefore, further research is required to test the proposed model across a representative national sample of hospital users, also taking into account how some organizational features could affect the relationship between OC and IS.

Despite some limitations, this paper contributes to the existing research on public-private differences within healthcare organizations by providing new empirical evidence of a link between the OC and IS.
